# Evidence for a Crucial Role of Paneth Cells in Mediating the Intestinal Response to Injury

**DOI:** 10.1002/stem.1326

**Published:** 2013-01-17

**Authors:** Lee Parry, Madeleine Young, Fatima El Marjou, Alan R Clarke

**Affiliations:** a School of Bioscience Biosciences, Cardiff UniversityCardiff, United Kingdom; b Institut Curie26 rue d'Ulm, Paris, France

**Keywords:** Intestine, Stem cells, Paneth cells, Genetically modified animals

## Abstract

The identification of the intestinal stem cell (ISC) markers *Lgr5* and *Bmi-1* has furthered our understanding of how they accomplish homeostasis in this rapidly self-renewing tissue. Recent work indicates that these markers identify a cycling *Lgr5*^+^ ISC which can be replaced by a quiescent Bmi-1^+^ ISC. Currently, there is little data on how these cells interact to control intestinal crypt homeostasis and regeneration. This interaction likely involves other differentiated cells within the niche as it has previously been demonstrated that the “stemness” of the *Lgr5* ISC is closely tied to the presence of their neighboring Paneth cells. To investigate this, we used two conditional mouse models to delete the transcription factor β-catenin within the intestinal crypt. Critically these differ in their ability to drive recombination within Paneth cells and therefore allow us to compare the effect of deleting the majority of active ISCs in the presence or absence of the Paneth cells. After gene deletion, the intestines in the model in which Paneth cells were retained showed a rapid recovery and repopulation of the crypt-villus axis presumably from either a spared ISC or the hypothetical quiescent ISCs. However, in the absence of Paneth cells the recovery ability was compromised resulting in complete loss of intestinal epithelial integrity. This data indicates that the Paneth cells play a crucial role within the in vivo ISC niche in aiding recovery following substantial insult. Stem Cells*2013;31:776–785*

## INTRODUCTION

The surface epithelia of the small intestine is a dynamic tissue which undergoes complete self-renewal every 3–4 days [1]. The epithelium comprises two basic repetitive structural units: (a) the crypts of Lieberkuhn which lie at the bottom of the mucosa and are continuous with (b) the villi, finger like projections which protrude into the lumen. This dynamic self-renewal originates from a population of ISCs located within the crypts which function as a stem cell “niche.” The proliferating and differentiating cells migrate upward onto the villi where they eventually die and are shed into the lumen.

For many years the identity of the ISCs was much sought after but the identification of two ISC markers in recent years has allowed progress to be made in the understanding of intestinal biology [2, 3]. The expression profile of these two ISC markers, *Bmi1* [3] and *Lgr5* [2], suggests that there may be two distinct ISC populations. The *Bmi1* expressing cells reside at the +4 position relative to the crypt bottoms. The *Lgr5* positive cells are the rapidly cycling columnar base cells (CBCs), with approximately 14 of these cells intermingled with the Paneth cells at the bottom of the crypt. Both these populations fulfill the stem cell characteristics of being self-renewing, multipotent, and essential for crypt maintenance [4]. However, the other stem cell hallmark of quiescence is currently only suggested for *Bmi1* cells [5] and is not a feature of the daily cycling CBCs. Based on these markers, it has been proposed that the crypt contains two stem cell populations. The first of these is an active/cycling stem cell population represented by the *Lgr5*-labeled CBCs [6]. The second is a *Bmi1* positive stem cell population at the +4 position which is capable of expanding and renewing the *Lgr5* population. Recently, this model has been supported by in vivo and in vitro data showing that, following either diphtheria toxin-mediated or radiation-induced killing of the *Lgr5* population, the *Bmi1* population can indeed function to replace the Lgr5 population [7, 8].

This concept of two populations of ISCs is further complicated by two recent reports, which demonstrate that self-renewal of the ISCs follows a pattern of neutral drift and elegantly identify a pool of equipotent stem cells that are regulated by its neighbors [9, 10]. This, together with the observation that *Lgr5* cells express the highest levels of *Bmi1*, suggests that *Bmi1* and *Lgr5* mark overlapping if not identical ISC populations [9, 11]. One of the crucial remaining questions is how these different stem cell compartments interact, especially during the process of crypt regeneration.

It is likely that aspects of the answer to this question will be found by an examination of the interactions between the ISCs and their neighboring cells within the crypt stem cell niche. Indeed the previous suggestion that the “stemness” of the CBCs is closely tied to the presence of their neighboring Paneth cells [9] has now been demonstrated in vitro and in vivo [12]. Evidence from previous studies in which depleting or deleting Paneth cells suggested they were dispensable in intestinal epithelial homeostasis have, upon closer examination shown that the *Lgr5* ISCs only exist where they can compete for essential niche signals provided by their specialized daughter Paneth cells [12].

The above studies examined the role of the Paneth cells in relation to presumably normal ISCs. The importance of Paneth cells in a situation where the ISC population is damaged is still unclear. One such circumstance is following loss of function of β-catenin, the conditional deletion of which has been reported to lead to different and conflicting outcomes [13, 14]. The first of these [14] used a tamoxifen (TAM) inducible variant of the Cre recombinase expressed under the control of the villin gene promoter to drive induction of Cre specifically in the intestinal epithelia [13, 15, 16] (vil-Cre-ER^T2^). Using this system, Fevr et al. [14] demonstrated rapid loss of transit amplifying cells, crypt structures, terminal differentiation of the ISCs, and loss of intestinal homeostasis and function upon deletion of β-catenin. In contrast, a separate study used the promoter element of the rat cytochrome P450A1 (*CYP1A1*) gene to drive *Cre* expression in a xenobiotic responsive manner to permit inducible gene deletion in the intestinal epithelia (*Ah-cre*) [13]. Ireland et al. [13] showed that deletion of β-catenin again reduced cell viability; however, the crypt-villus axis was subsequently rapidly repopulated with unrecombined cells expressing β-catenin and homeostasis was re-established. Given that long-term labeling studies have shown that both the *vil-Cre-ER*^*T2*^ and *Ah-cre* models induce recombination in the ISCs, the mechanisms underlying these different outcomes are unclear. One possibility is that the two systems differentially drive recombination within the stem cell population, such that in the *vil-Cre-ER*^*T2*^ system a greater proportion of the ISC population is recombined. An alternative possibility is that the *vil-Cre-ER*^*T2*^ model deletes *CatnB* in differentiated cells, which provide the ISC niche. In the latter scenario, the most obvious candidate here is the Paneth cell, as this has previously been shown to be spared by *Ah-cre* [13]. To directly address these possibilities, we have confirmed conditions in which both systems deliver the same level of stem cell recombination and have subsequently determined the additional effect of recombining within the Paneth cell population using the *vil-Cre-ER*^*T2*^ system. Analysis of this data indicates that the Paneth cells are critically sensitive to β-catenin loss, and that their loss critically compromises the capacity of intestinal crypts to recover following insult.

## MATERIALS AND METHODS

### Animal Models

The alleles for the *vil-Cre-ER*^*T*2^[17], *Ah-cre* [13], floxed β-catenin (*CatnB*^*flox*^) [18], and Rosa26R-*lacZ* [19] have been described previously. All experiments were performed under the authority of a U.K. project license, which is issued after a local ethical review process. For induction of *Ah-cre*, the mice received intraperitoneal (i.p.) injections of 80 mg/kg β-napthoflavone (βNF) (Sigma, U.K., http://www.sigma.com) dissolved in corn oil (10 mg/ml) at frequencies stated. For induction of the *vil-Cre-ER*^*T2*^, the mice received i.p. injections of 80 mg/kg TAM unless otherwise stated (Sigma, U.K.) dissolved in corn oil (10 mg/ml) at doses stated. Control experiments were performed by inducing mice which only contained the appropriate *Cre* transgene and *lacZ* reporter, referred to as wild type (WT).

### Relative Quantitation Analysis

The following methods were all performed according to manufacturer's instructions unless otherwise stated. For analysis of either the levels of *CatnB* allele recombination or gene expression in the intestinal epithelia, three to five mice from each control and experimental group were harvested. DNA or RNA was extracted either from a 0.5 cm portion of small intestine taken 2–3 cm distally from the stomach and stored at −80°C in RNAlater (Sigma, U.K.) or from crypt epithelia extracted from a whole small intestine [20]. Genomic DNA was extracted using the QiaAMP kit according to manufacturers' instructions and approximately 100 ng was used for quantitative polymerase chain reaction (qPCR). Total RNA was extracted using the RNeasy kit (Qiagen, U.K., http://www.qiagen.com) and DNase treated using the Turbo DNase kit (Ambion, U.K., http://www.ambion.com). Complimentary DNA was transcribed from 1 μg of RNA using random hexamers (Promega, U.K., http://www.promega.com) and the Superscript III (Invitrogen, U.K., http://www.invitrogen.com) kits. For relative quantitation, all samples were run in duplicate on the StepOnePlus PCR machine using Fast Sybr Green master mix (Applied Biosystems, U.K., http://www.appliedbiosystems.com) or Taqman Univeral Mastermix II (Applied Biosystems). The threshold cycle (*C*_*t*_) values of each gene analyzed were normalized to a reference gene. For qPCR on genomic DNA, *C*_*t*_ values were normalized against the single copy gene *ApoB*, for expression analysis (qRT-PCR) *C*_*t*_ values were normalized against the *ActB* gene. Differences between groups were assessed using the 2^−ΔΔCT^ method [21]. One-tailed Mann–Whitney *U* (M.W.) tests were performed on the Δ*C*_*t*_ values to determine significant (*p* = <.05) differences between groups [22]. Relative quantification was performed using the oligonucleotide sequences indicated in Table 1.

**Table 1:** Details of primers and assay used for expression and genomic quantitative analysis

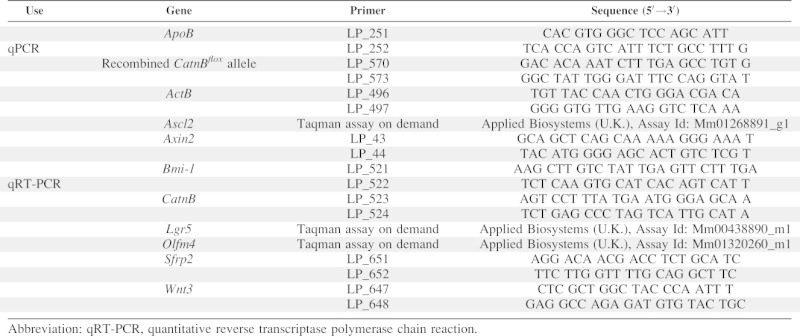

Abbreviation: qRT-PCR, quantitative reverse transcriptase polymerase chain reaction.

### Reporter Visualization and Immunohistochemistry

Whole intestines were mounted and opened along the mesenteric line and stained for *LacZ* activity [17]. Expression patterns were determined from at least three animals per experimental group using a standard dissecting microscope. The total numbers of stained crypts were counted within the 2–5 cm region distally from the stomach using imageJ software (http://rsbweb.nih.gov/ij/).

For immunohistochemistry (IHC), tissue was fixed in ice cold 10% formalin and processed into wax blocks by conventional means. Section were cut at 5 μm, dewaxed, and rehydrated into phosphate buffered saline. Staining was performed using the Envision+ mouse or rabbit kit (Cat No# K4007 & K4011, Dako, U.K., http://www.dako.com) according to manufacturer's instructions. To detect loss of β-catenin protein, we used a mouse Mab anti-β-catenin antibody (Cat No#610154, BD/Transduction Laboratories, U.K., http://www.bdbiosciences.com) at 1:200, for Paneth cell detection, we used a rabbit pAb anti-lysozyme antibody (Cat No# RB-372, Neomarkers/Labvision, USA, http://www.labvision.com) at 1:200, for proliferation, Ki67 mouse Mab at 1:200 (Cat No# VP-K452, Vector Laboratories, U.K., http://www.vectorlabs.com), and for apoptosis detection, we used a cleaved caspase 3 (Cat No# 9661, Cell Signaling Technologies, U.K., http://www.cellsignal.com) at 1:200. For visualization using immunofluorescence, sections were labeled using Alexafluor 488 and 594 secondary antibodies (Cat No# A11001 and A11072, Invitrogen) at 1:500. Detection of *Olfm4* RNA by in situ hybridization was performed using protocols previously described [23–25]. The apoptotic and mitotic index were scored from H&E sections as previously described [26, 27]. Numbers of crypts were counted on 5–10 H&E stained cross-sections from each mouse. Paneth cell number was determined by counting the numbers of cells stained positive for lysozyme in a total of 25 crypts taken from at least three different sections for each mouse.

## RESULTS

### Identification of a Regime that Delivers Equivalent Levels of Stem Cell Recombination in the *Ah-Cre* and *vil-Cre-ER^T2^* Systems

**Figure 1:**
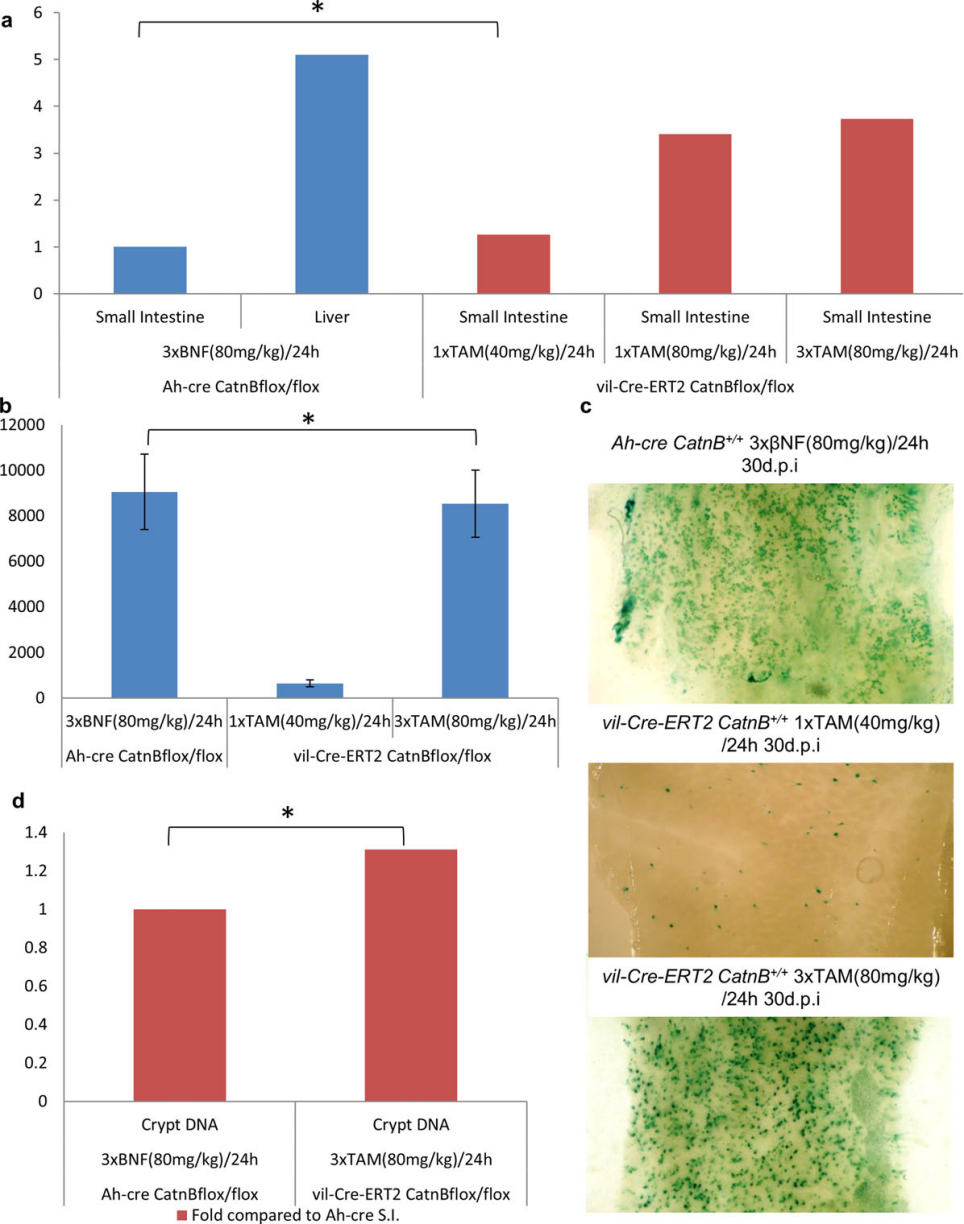
Comparison of the specificity and efficiency of *Cre/Lox* recombination within the intestinal epithelia using the *Ah-cre* and *vil-Cre-ER*^*T2*^ systems (*, *p* > .05). (**A**): Results of quantitative polymerase chain reaction (qPCR) for detection of fold change of the recombined *CatnB*^*flox*^ allele in the small intestine using different induction regimes. (**B**): Quantification of *LacZ* positive stained crypts 30 d.p.i. using regimes indicated. (**C**): Whole mount small intestine showing *LacZ* stained crypts within the small intestine. (**D**): Results of qPCR for detection of CatnB^*flox*^ allele within the intestinal crypt epithelia using different induction regimes. Abbreviations: βNF, β-napthoflavone; TAM, tamoxifen.

We first defined the level of recombination driven in the *Ah-cre* system both within the whole intestine and within the stem cell compartment. To define overall recombination levels, we induced *Ah-cre CatnB*^*flox/flox*^ mice with 3 × i.p./24 hour injections of βNF and collected tissues at 1-day postinduction (d.p.i). We then quantified the presence of recombined *CatnB*^*flox*^ alleles in the genomic DNA from whole intestine as a surrogate marker for the numbers of cells recombined. qPCR analysis showed that for *Ah-cre CatnB*^*flox/flox*^ mice average ΔΔ*C*_*t*_ value was 2.28 in small intestine compared to −0.07 in the liver, a tissue previously shown to have high levels of recombination (Fig. 1A). To determine the efficiency of recombination in the ISC, we induced *Ah-cre Rosa26R-lacZ CatnB*^*wt/wt*^ mice with 3 × i.p./24 hour injections of βNF and analyzed intestines at 30 d.p.i. The number of *LacZ* positive crypts were counted on intestinal whole mounts as a marker of stem cell recombination and established a baseline of an average 9,267 recombined crypts (representing recombined ISCs) (Fig. 1B, 1C).

We next compared the *Ah-cre* baseline with three different induction regimes in the *vil-Cre-ER*^*T2*^ system: 3 × i.p. injections of TAM (80 mg/kg)/24 hour, 1 × i.p. injection of TAM (80 mg/kg)/24 hour, and 1 × i.p. injection of TAM (40 mg/kg)/24 hour. The average ΔΔ*C*_*t*_ value for mice recombining using the highest dose was 0.38, indicating a significant 3.53 (*p* < .404 M.W.)-fold greater amount of the recombined *CatnB*^*flox*^ allele compared to the *Ah-cre* baseline (Fig. 1A). This result is most likely a reflection of the *vil-Cre-ER*^*T2*^ system, which recombines in all epithelial cells of the crypt and villus, in contrast to the *Ah-cre* system which only recombines in the ISC niche and transit amplifying compartment of the crypt. However, we cannot exclude the possibility that some of the difference observed is due to each *Cre* system having different recombination efficiency at the *CatnB*^*flox*^ allele. The intermediate regime delivered a *C*_*t*_ value of 0.51, which was not significantly different to the highest dose (Fig. 1A). At the lowest dose, the average Δ*C*_*t*_ value was 1.94, indicating a 1.1-fold increase in presence of recombined *CatnB*^*flox*^ allele compared to the *Ah-cre* baseline, which was not significantly different (Fig. 1A). For the highest and lowest dose regimes, we then determined the level of ISC recombination (Fig. 1B, 1C). The high regime delivered an average of 8,535 recombined crypts indicating ISC recombination rates equivalent to the *Ah-cre* baseline. The lowest regime showed a greatly reduced rate with 646 recombined crypts. These experiments therefore established that the high-dose *vil-Cre-ER*^*T2*^ regime delivered the same level of ISC recombination as the *Ah-cre* regime. Notably, this level of induction results in elevated overall levels of recombination compared to *Ah-cre*, which presumably reflects the previously reported higher levels of recombination in the villus in the *vil-Cre-ER*^*T2*^ system. In further support of this, analysis of purified crypts from induced mice showed that the higher *vil-Cre-ER*^*T2*^ induction regime delivered equivalent levels of recombination to the *Ah-cre* baseline (Fig. 1D).

### Ah-cre and vil-Cre-ER^T2^ Driven Deletion of β-Catenin Results in Different Small Intestine Phenotypes

**Figure 2:**
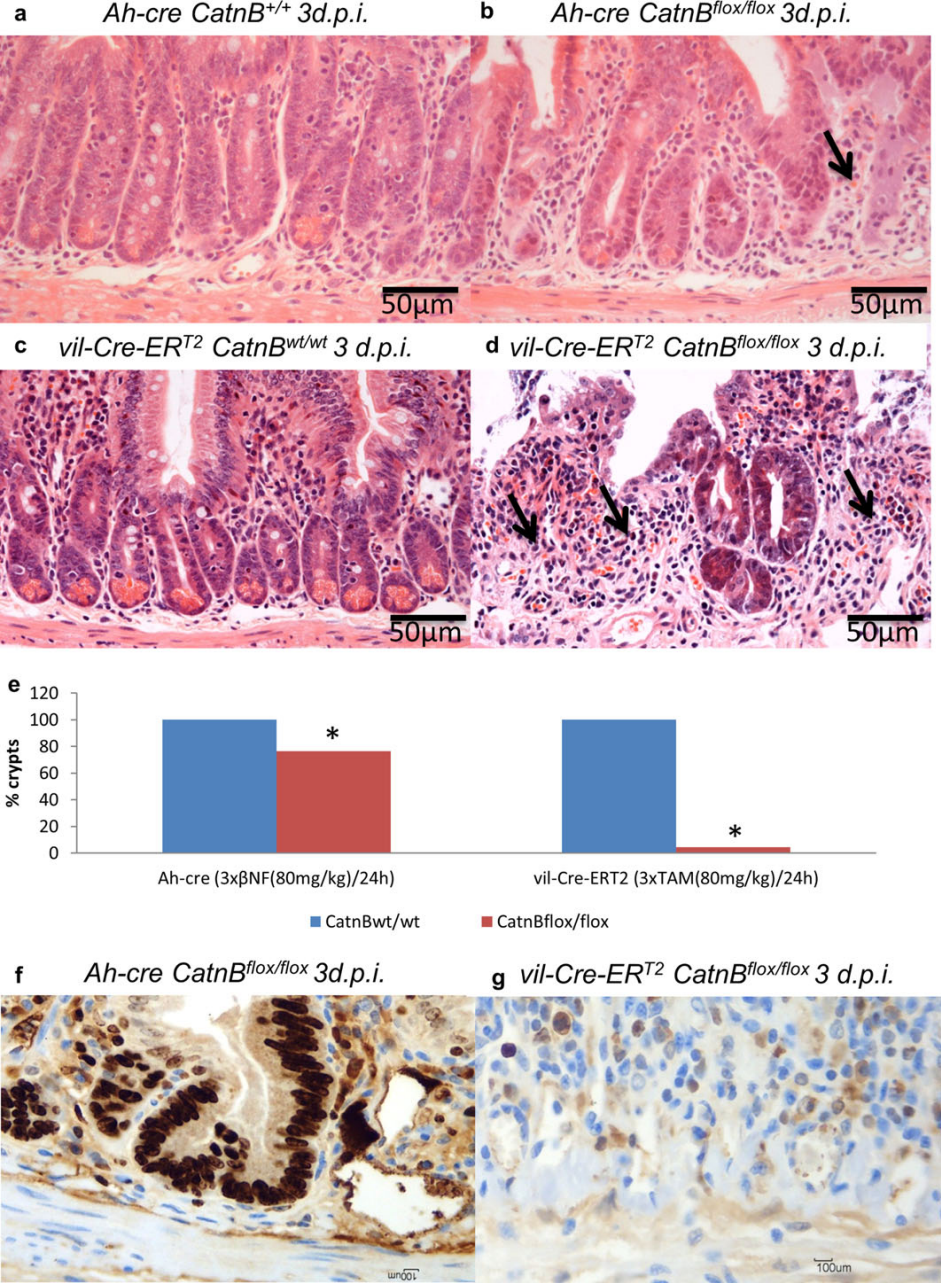
Comparison of the *Ah-cre* and *vil-Cre-ER*^*T2*^ systems for conditionally deleting β-catenin within the small intestine epithelia. (**A–D**): H&E stained cross-sections of the small intestine, arrow indicate areas of crypt loss. (**E**): Quantification of the percentage of intestinal crypts lost 30 days after deletion of β-catenin (*, *p* < .01 M.W.) (**F, G**): Ki-67 staining indicating proliferating cells at 3 d.p.i. Abbreviations: βNF, β-napthoflavone; TAM, tamoxifen.

We next assessed whether the different phenotypes previously observed were due to the use of differently targeted *CatnB*^*flox*^ alleles. To establish this, we used the *Ah-cre* and *vil-Cre-ER*^*T2*^ mice to delete the same *CatnB*^*flox*^ [18] allele using the regimes characterized above that deliver equivalent levels of ISC recombination. In both models, deletion of *CatnB* from the intestinal epithelium generated phenotypes similar to the previous reports. *Ah-cre* mice (3 × i.p. βNF (80 mg/kg)/24 hour) showed no obvious signs of distress or reduction of survival by 30 d.p.i. and partial loss of crypts at 3 d.p.i. compared to WT (Fig. 2A, 2B, 2E). The *vil-Cre-ER*^*T2*^ mice (3 × i.p. TAM (80 mg/kg)/24 hour) became moribund by day 4–5 and had to be euthanized and at 3 d.p.i. mice showed destruction of the crypt/villus axis compared to WT (Fig. 2C–2E).

### Vil-Cre-ER^T2^ Driven β-Catenin-Deficient Intestines Are Unable to Repopulate like Ah-cre

**Figure 3:**
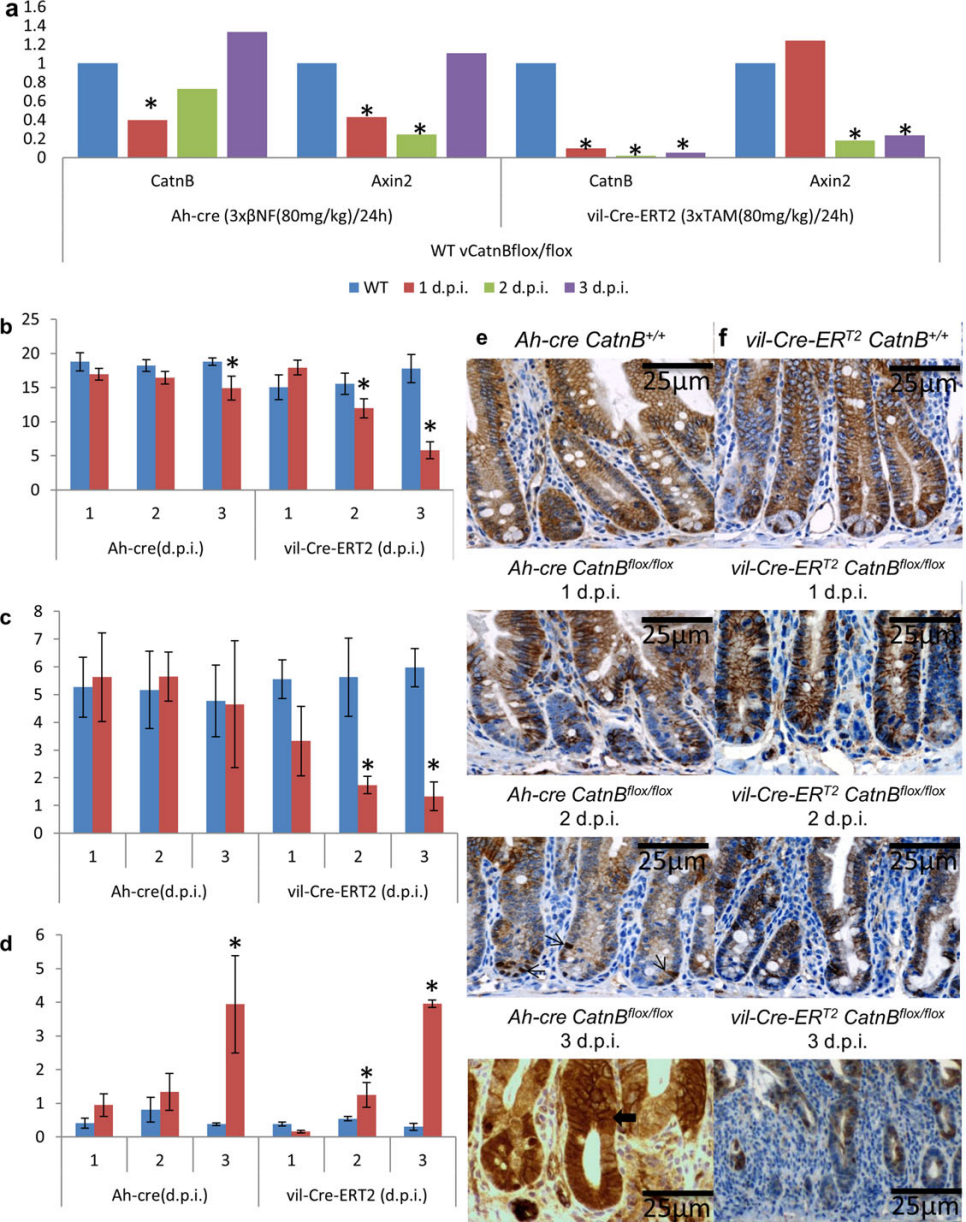
Characterization of the onset of phenotype when using *Ah-cre* and *vil-Cre-ER*^*T2*^ systems for conditionally deleting β-catenin in a similar number of intestinal stem cells within the small intestine epithelia. (**A**): Expression analysis of genes highlighting the phenotype of loss of *CatnB* in the small intestine epithelia (*, *p* < .01 M.W.) in comparison to wild-type (WT) controls. Quantification of the cellular phenotype between WT (blue) and *CatnB*^*flox*^ (red) mice at each time point (**B**) crypt height, (**C**) mitosis, and (**D**) apoptosis (*, *p* < .05 M.W.). (**E, F**): Immunohistochemistry demonstrating pattern of β-catenin loss and regeneration in *Ah-cre* but not *vil-Cre-ER*^*T2*^ mice (→ indicate cells with nuclear β-catenin; 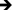 indicates β-catenin deficient cells migrating up on to villus). Abbreviations: βNF, β-napthoflavone; TAM, tamoxifen.

To understand the reasons for the survival of Ah-cre mice in contrast to the vil-Cre-ER^T2^ mice, we characterized the epithelia after β-catenin deletion. Using the LacZ reporter as a surrogate marker both models showed that after 3 d.p.i. there was a complete loss of all β-catenin-deficient cells compared to controls (data not shown). This was confirmed by expression analysis which demonstrated the deletion of the *CatnB* gene and alteration of the canonical Wnt pathway target *Axin2* (Fig. 3A) compared to appropriate WT controls. For *Ah-cre*, *CatnB* expression fold changes were as follows: 1d/0.4*, 2d/0.73, and 3d/1.3, and this trend was reflected by Axin2: 1d/0.43*, 2d/0.24*, and 3d/1.1 (*, *p* ≤ .01 M.W.). The return to WT expression levels indicated that the epithelium had been repopulated with WT cells. In comparison there was no indication the *vil-Cre-ER*^*T2*^ mice had repopulated as the fold changes were for *CatnB*: 1d/0.1*, 2d/0.02*, and 3d/0.05* and *Axin2*: 1d/1.2, 2d/0.18*, and 3d/0.23* (*p ≤ .01 M.W.).

**Figure 4:**
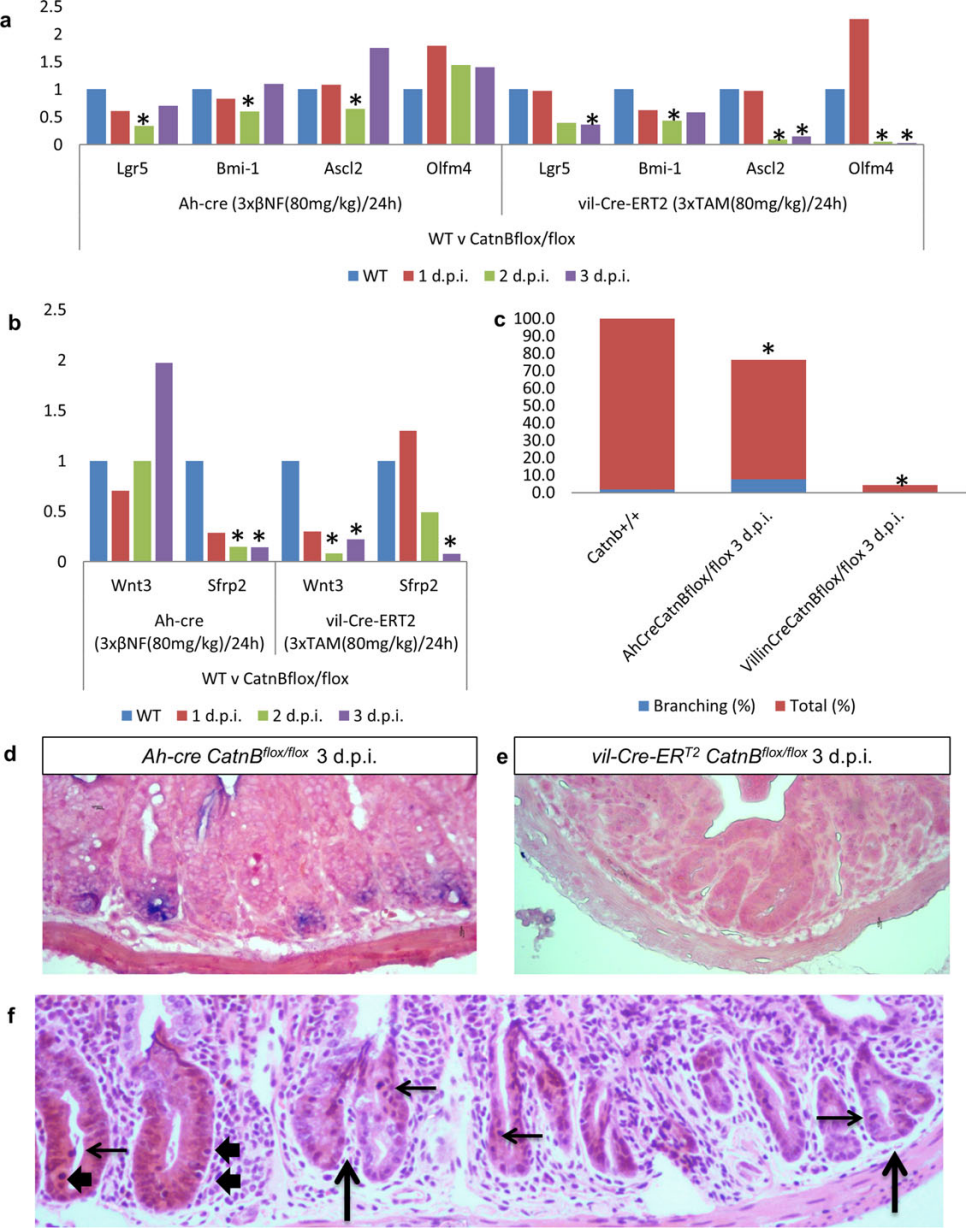
Comparison of the intestinal stem cell (ISC) characteristics using *Ah-cre* and *vil-Cre-ER*^*T2*^ systems for conditionally deleting β-catenin within the small intestine epithelia. Expression analysis of (**A**) ISC marker genes and (**B**) Wnt regulators highlighting the phenotype of loss of *CatnB* in the small intestine epithelia (*, *p* < .01 M.W.) in comparison to wild-type (WT) controls. (**C**): Cellular phenotype indicating % of crypts (red) and the % of branching crypts (blue) (*, *p* < .01 M.W.) in comparison to WT controls. (**D, E**): In situ hybridization for expression of the ISC marker *Olfm4*. (**F**): H&E stained cross-section of small intestine from *Ah-cre CatnB*^*flox*^ at 3 d.p.i.; → indicates mitotic figures, ↑ indicates branching crypts, and 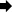 indicates apoptotic bodies. Abbreviations: βNF, β-napthoflavone; TAM, tamoxifen.

The repopulation in *Ah-cre* mice compared to *vil-Cre-ER*^*T2*^ was further demonstrated by β-catenin expression patterns (Fig. 3E, 3F). The *Ah-cre* and *vil-Cre-ER*^*T2*^*CatnB*^*flox/flox*^ mice show no apparent loss of β-catenin at 1 d.p.i. compared to a WT mouse. At 2 d.p.i., *vil-Cre-ER*^*T2*^ mice mice show a loss of β-catenin staining in the crypt and villus region. In comparison, the loss is confined to the crypts in the *Ah-cre* mice, however, these crypts show evidence of β-catenin nuclear localization in single cells near the crypt base. By 3 d.p.i., the β-catenin-deficient crypt cells in the *Ah-cre* mice have migrated up onto the villus and been replaced with WT cells, in contrast the majority of the crypts in the *vil-Cre-ER*^*T2*^ mice have been lost, however, the few remaining crypts show normal β-catenin localization patterns. These remaining crypts, however, are negative for the proliferation marker Ki-67 and ISC marker *Olfm4* unlike the crypts in the *Ah-cre* mice (Figs. 2F, 2G and 4D, 4E). Further evidence of recovery in the *Ah-cre* was observed in apoptosis, crypt length, crypt numbers, mitosis, and proliferation levels over 1–3 d.p.i. (Figs. 3B–3D and 4F). In both sets of mice there was a trend of decreasing crypt length by 3 d.p.i., *Ah-cre* mice showed a 10.8% loss in crypt length compared to a 67.3% (*p* = <.05) decrease in *vil-Cre-ER*^*T2*^ mice. However, both *Ah-cre* and *vil-Cre-ER*^*T2*^ mice showed similar increases in apoptosis within the crypts but a reduction in mitosis was only observed in the *vil-Cre-ER*^*T2*^ mice with a 77.8% decrease in mitotic figures. Recovery was also supported by the examination of expression levels of ISC markers and Wnt signaling genes when compared to the appropriate WT controls. Expression levels of the stem cell markers *Ascl2*, *Bmi-1*, *Lgr5*, and *Olfm4* recovered to WT levels in *Ah-cre CatnB*^*flox/flox*^ by 3 d.p.i. but not the *vil-Cre-ER*^*T2*^*CatnB*^*flox/flox*^ mice (Fig. 4A). Whereas, the increase in expression of the Wnt ligand, *Wnt3*, correlated with a decrease in the Wnt inhibitor *Sfrp2* in *Ah-cre* mice by 3 d.p.i. but not the *vil-Cre-ER*^*T2*^*CatnB*^*flox/flox*^ mice (Fig. 4B). This is consistent with an activation of the Wnt pathway to drive repopulation. Further, this recovery of crypt numbers and repopulation in the *Ah-cre* mice correlates with a significant increase in the proportion of crypts undergoing branching, a surrogate marker for crypt fission, which was absent in the vil-*Cre-ER*^*T2*^ mice (Fig. 4C, 4F).

### Vil-Cre-ERT2 but Not *Ah-Cre* Deletes β-Catenin in the Paneth Cells Leading to Their Death

**Figure 5:**
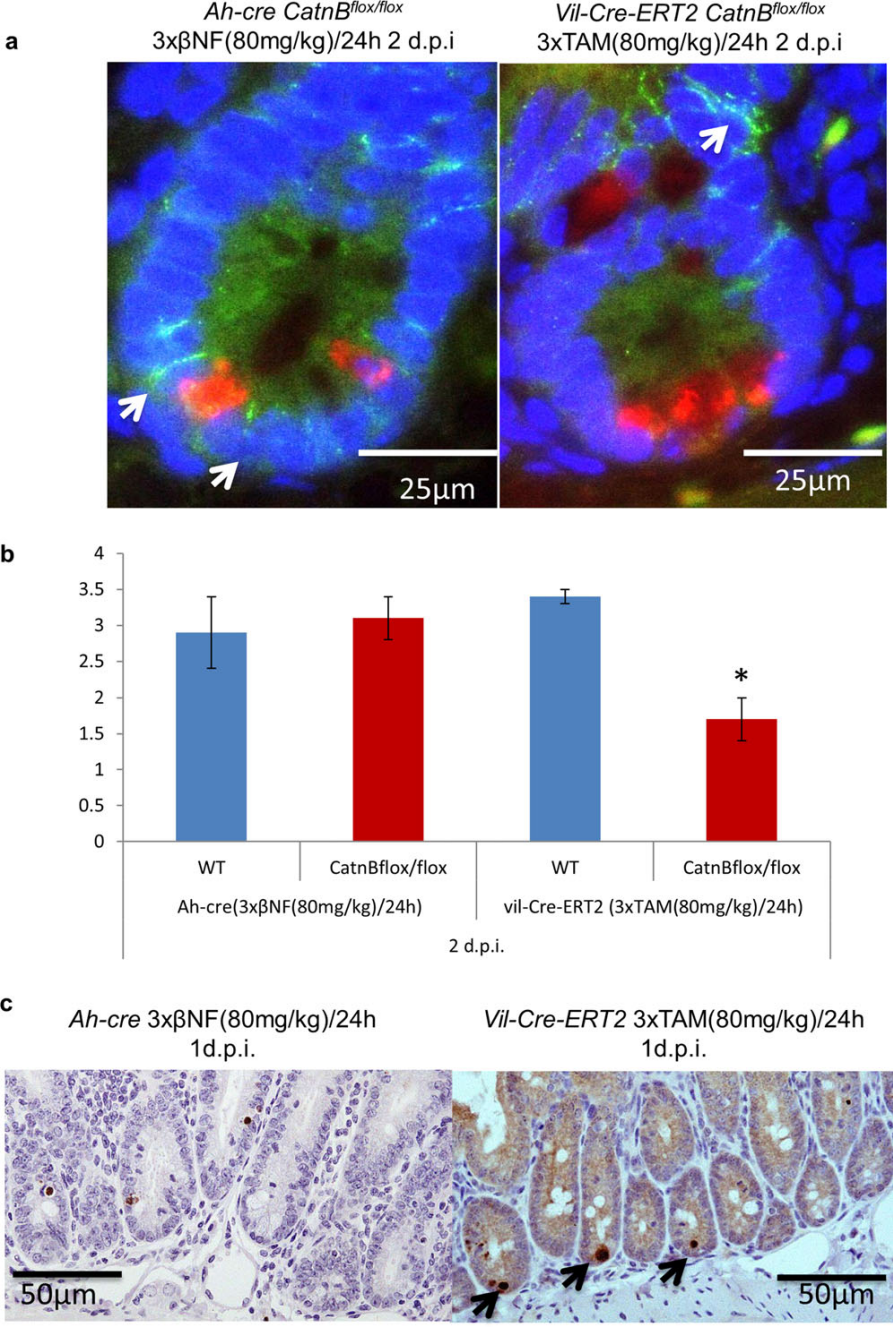
Characterization of the Paneth cells after β-catenin deletion using the *Ah-cre* and *vil-Cre-ER*^*T2*^ systems. (**A**): Immunofluorescence to indicate *CatnB* (green) deletion only occurs in the Paneth cells (red) using the *vil-Cre-ER*^*T2*^ system. (**B**): Quantification of the number of Paneth cells present 2 days after deletion of β-catenin (*, *p* < .04 M.W.). (**C**): IHC staining for cleaved caspase 3 indicating apoptotic cells at the base of the crypt, where the Paneth cells reside, in the *vil-Cre-ER*^*T2*^ but not the *Ah-cre* mice (→ indicates position of Paneth cells). Abbreviations: βNF, β-napthoflavone; TAM, tamoxifen; WT, wild type.

Using immunofluorescence, we showed that *vil-Cre-ER*^*T2*^ but not *Ah-cre* recombines within the Paneth cell population (Fig. 5A), as has been previously reported. To define the effects of deleting β-catenin in these cells, we scored the presence of Paneth cells, after IHC staining with an antibody against lysozyme at 2 d.p.i. There was a significant reduction in Paneth cell number between *vil-Cre-ER*^*T2*^*CatnB*^*flox/flox*^ and *vil-Cre-ER*^*T2*^*CatnB*^+/+^ control mice (*p* < .048 M.W.) which was not observed with *Ah-cre CatnB*^*flox/flox*^ mice (Fig. 5B). The survival of the Paneth cells in the *Ah-cre* mice was further supported by an increase in expression of the Paneth cell expressed Wnt ligand *Wnt3*, which was absent in *vil-Cre-ER*^*T2*^ mice (Fig. 4B). We next used IHC to detect cleaved caspase 3 as a marker of apoptosis. After induction for the deletion of β-catenin, Paneth cells positive for cleaved caspase 3 were readily detected in the *vil-Cre-ER*^*T2*^ mice but rarely observed in the *Ah-cre* mice (Fig. 5C) indicating that β-catenin is critical for the survival of the Paneth cells.

## DISCUSSION

Recently, two populations of cells have been proposed to contain the ISC. One is marked by *Bmi-1* expression and is found at position four in the crypt; the second is marked by *Lgr5* expression and is located at the base of the crypt. It currently remains unclear how cells transit between these populations, although it has been demonstrated that the *Bmi-1* positive population can recapitulate the *Lgr5* population. One interpretation of this data is that the *Lgr5* population is an “active” stem cell pool, which can be replenished from a more quiescent *Bmi-1* pool of cells. This active *Lgr5* population appears to be reliant on interactions with the Paneth cells within the crypt, which have been postulated to play a “nurse” role in maintaining stemness. We sought to further understand this interaction by introducing a damaging mutation into the ISC population in the presence or absence of Paneth cells. We selected deletion of β-catenin as the damaging mutation, as previous studies have shown remarkably diverse phenotypic effects of β-catenin loss dependent on the *Cre* transgene being used. Thus, deletion mediated by *vil-Cre-ER*^*T2*^ leads to catastrophic loss of the epithelium and animal death as opposed to *Ah-cre* mediated deletion, in which there is rapid repopulation by WT cells and no lethality.

These observations lead to two hypotheses. First, that the difference seen between the two systems is a direct reflection of differing recombination rates within the ISC compartment (either *Bmi-1* or *Lgr5* positive). Second, that the difference arises as a consequence of differing recombination rates in differentiated cells, such as the Paneth cells. To address these possibilities, we first defined dosing regimens that would deliver equivalent levels of ISC recombination using either *Cre* system. Having established this baseline, we next ensured that the differences in phenotypes previously observed were not due to technical differences associated with use of different *CatnB*^*flox*^ alleles. We therefore performed repeat experiments with the two *Cre* strains and the same *CatnB*^*flox 18*^ allele. For both *Cre* transgenes, we observed a similar phenotype to that previously reported in that we observed intestine survival and repopulation using *Ah-cre* but crypt loss and death using *vil-Cre-ER*^*T2*^. These data show that the phenotypic differences are not attributable to varying levels of functional ISC recombination.

It has previously been shown that the major difference in cell type targeting within the crypt between these two systems is that *Ah*-*cre* does not drive recombination within the Paneth cells [13, 17]. However, it does drive recombination in goblet cells, another intestinal epithelium secretory cell, where deletion of *CatnB* results in a loss of goblet cells [13]. We again confirm here that the Paneth cells are spared in the *Ah-cre* model and go on to show that loss of β-catenin within the *vil-Cre-ER*^*T2*^ model leads to Paneth cell death. One interpretation of our data is that this is the critical difference between the two models, with loss of Paneth cells having a profound effect on the capacity to regenerate the crypt. This interpretation is supported by recent work demonstrating that when using the *vil-Cre-ER*^*T2*^ model to delete *Math-1* all Paneth cells were lost but crypt architecture was otherwise unperturbed, as other *Math-1*-deficient cells expressed the necessary Wnts [28] whereas other models deleting only Paneth cells show crypt loss [12]. The alternate explanation is that *vil-Cre-ER*^*T2*^ more efficiently targets a potential reserve ISC population. As our data clearly shows that, where repopulation occurs, it always occurs from an unrecombined population, indicating that *Ah-cre* does indeed spare a “reserve” population of stem cells. The identity of this population is unknown and it is of note that there is no increase in the expression of the *Bmi-1* gene, as cells which express this gene have been proposed to contain a “reserve ISC population.” However, the increase in crypt fission in *Ah-cre* mice does suggest that the reserve population originates from within the ISC niche. Whether this reserve population represents unrecombined ISCs or dedifferentiated ISC daughter cells remains to be seen.

## CONCLUSION

In summary, we have explored two models of *Cre*-mediated intestinal epithelial deletion that have markedly different consequences for intestinal regeneration. We show that the differences we observe do not arise as a consequence of differential recombination rates within the functional ISC. Future work will need to clarify between the three possibilities that are consistent with our data: first, that the Paneth cell is critical as a nurse cell during crypt regeneration; second that the reserve ISC is differentially targeted by the *vil-Cre-ER*^*T2*^ and *Ah-cre* systems, and finally that both the reserve ISC and Paneth cell compartments are differentially recombined and both contribute to the observed phenotypes.
